# Phenol-Soluble Modulin α Peptide Toxins from Aggressive *Staphylococcus aureus* Induce Rapid Formation of Neutrophil Extracellular Traps through a Reactive Oxygen Species-Independent Pathway

**DOI:** 10.3389/fimmu.2017.00257

**Published:** 2017-03-09

**Authors:** Halla Björnsdottir, Agnes Dahlstrand Rudin, Felix P. Klose, Jonas Elmwall, Amanda Welin, Marios Stylianou, Karin Christenson, Constantin F. Urban, Huamei Forsman, Claes Dahlgren, Anna Karlsson, Johan Bylund

**Affiliations:** ^1^Department of Oral Microbiology and Immunology, Institute of Odontology, Sahlgrenska Academy at University of Gothenburg, Gothenburg, Sweden; ^2^The Phagocyte Research Laboratory, Department of Rheumatology and Inflammation Research, Institute of Medicine, Sahlgrenska Academy at University of Gothenburg, Gothenburg, Sweden; ^3^Antifungal Immunity Group, Department of Clinical Microbiology, Umeå University, Umeå, Sweden

**Keywords:** community-acquired methicillin-resistant *Staphylococcus aureus*, PSM, NETs, ROS, FPR2, MPO, neutrophil elastase, Papillon-Lefèvre syndrome

## Abstract

Neutrophils have the ability to capture and kill microbes extracellularly through the formation of neutrophil extracellular traps (NETs). These are DNA and protein structures that neutrophils release extracellularly and are believed to function as a defense mechanism against microbes. The classic NET formation process, triggered by, e.g., bacteria, fungi, or by direct stimulation of protein kinase C through phorbol myristate acetate, is an active process that takes several hours and relies on the production of reactive oxygen species (ROS) that are further modified by myeloperoxidase (MPO). We show here that NET-like structures can also be formed by neutrophils after interaction with phenol-soluble modulin α (PSMα) that are cytotoxic membrane-disturbing peptides, secreted from community-acquired methicillin-resistant *Staphylococcus aureus* (CA-MRSA). The PSMα-induced NETs contained the typical protein markers and were able to capture microbes. The PSMα-induced NET structures were disintegrated upon prolonged exposure to DNase-positive *S. aureus* but not on exposure to DNase-negative *Candida albicans*. Opposed to classic NETosis, PSMα-triggered NET formation occurred very rapidly, independently of ROS or MPO, and was also manifest at 4°C. These data indicate that rapid NETs release may result from cytotoxic membrane disturbance by PSMα peptides, a process that may be of importance for CA-MRSA virulence.

## Introduction

*Staphylococcus aureus* is an important pathogen that is the leading cause of bacterial infections worldwide (1). The bacteria is both commensal, colonizing about one-third of humans (2, 3), and a pathogen that causes a variety of infection from superficial skin infections to lethal systemic infections. Among the important pathogenic strains are the community-acquired methicillin-resistant *S. aureus* (CA-MRSA) strains that are both antibiotic resistant and have acquired additional virulence factors that enable them to cause especially aggressive infections, even in healthy individuals (4).

Neutrophils are essential cells in fighting bacterial infections in general, not least *S. aureus* infections; they have the ability to neutralize microbes intracellularly through phagocytosis or extracellularly through the formation of neutrophil extracellular traps (NETs). These are extracellular structures consisting of the neutrophils’ own DNA covered in intracellular proteins, such as myeloperoxidase (MPO), neutrophil elastase (NE), and histones (5). NETs can be formed after exposure to microbes, microbial products, cytokines, or synthetic chemicals [reviewed in Ref. (6)] and is mainly considered to be a programmed cellular suicide. The formation of NETs has been characterized extensively *in vitro* by using phorbol myristate acetate (PMA); this type of NETosis is completely dependent on the production of reactive oxygen species (ROS) through the neutrophil NADPH oxidase (7), and these ROS require further transformation by myeloperoxidase (MPO) (8, 9) inside granules (10). Furthermore, the ROS-MPO-dependent NET formation is preceded by a coordinated series of cellular events, and although the mechanistic details are not completely understood, it is established that the nuclear matter is decondensed before NETs are released and that the entire process takes several hours (7, 11, 12). This type of NET formation is often called suicidal NETosis as it leads to loss of plasma membrane integrity and neutrophil death (13). In addition to PMA, suicidal NETosis has been described to occur after treatment of neutrophils with bacteria (7, 12) and fungi (8, 14). A distinct type of NET formation, “vital NETosis,” whereby the anucleated neutrophils remain intact and functional after throwing out their DNA has also been described after interactions with certain bacteria and/or bacterial products (15–18).

It is known that *S. aureus per se* can induce NET formation (7) and also that factors secreted from *S. aureus* can induce NET formation (15, 16, 19). The CA-MRSA strains secrete high levels of phenol-soluble modulins (PSMs), peptides that are important virulence factors for these strains (20). These peptides can be divided into three groups, the shorter α-types (PSMα 1–4), the longer β-types (PSMβ 1–2), and the δ-toxin (an α-like peptide) (20). We and others have previously shown that low nanomolar concentrations of PSMα peptides attract and activate human neutrophils by binding to the chemoattractant receptor formyl peptide receptor (FPR) 2 (21, 22). At higher (high nanomolar to low micromolar) concentrations, the PSMα peptides are cytotoxic to neutrophils and able to permeabilize the plasma membrane (20), with an apparent preference for apoptotic membranes (22).

In this study, we found that micromolar concentrations of PSMα peptides trigger neutrophils to form extracellular traps that were morphologically and functionally indistinguishable to the classic NETs induced by PMA. In contrast to PMA-induced NET formation, the PSMα-induced process was very rapid, occurring within 5–10 min after stimulation and did not require neutrophil ROS, MPO, or NE activity. In addition, PSMα-triggered NET formation was independent of FPR2 and took place also at 4°C, albeit at a slower pace, indicating that active cell signaling was not needed. Our data thus demonstrate a novel type of NET formation induced by PSMα peptides that may be of importance for *S. aureus*, especially CA-MRSA strains, virulence.

## Materials and Methods

### Isolation of Human Neutrophils

Peripheral blood neutrophils were isolated from 1-day-old buffy coats from healthy blood donors or from peripheral blood from patients and healthy controls. Neutrophil separation was performed as first described by Boyum (23). Briefly, after dextran, sedimentation at 1 × *g* the suspension was centrifuged on Ficoll-Paque, and the remaining erythrocytes were lysed by hypotonic treatment. The neutrophils were then washed in Krebs Ringer phosphate buffer (KRG) and finally resuspended in KRG supplemented with Ca^2+^ (1 mM) and kept on melting ice until used. Buffy coats were obtained from the Sahlgrenska University Hospital blood bank after deidentification, and according to the Swedish legislation section code 4§ 3 p SFS2003:460, no informed consent is needed. Peripheral blood from one MPO-deficient individual, one patient with Papillon-Lefèvre syndrome, and healthy controls were obtained after written informed consent, and the study was approved by the Regional Ethical Review Board in Gothenburg, Sweden.

### A375 Melanoma Cell Line

The human melanoma cell line A375 was a kind gift from Professor Jonas A. Nilsson (Sahlgrenska Cancer Center, University of Gothenburg, Sweden). The cells were cultured in DMEM with 10% FCS, 100 U/ml penicillin, and 100 μg/ml streptomycin at 37°C and 5% CO_2_. Before being used experimentally, the cells were detached from the flasks’ surface by incubation with 0.25% trypsin in 37°C for 10 min followed by washing and resuspension in RPMI culture media.

### PSMα Peptides

PSMα2 (fMGIIAGIIKFIKGLIEKFTGK), PSMα3 (fMEFVAKLFKFFKDLLGKFLGNN), and the PSMα2 variants (Table 1) in their formylated forms were synthesized by American Peptide Company or EMC microcollection. The stocks were made in dimethyl sulfoxide and further diluted in the same medium as used in the experiments.

**Table 1:** **Peptide sequences and receptor preferences of PSMα2**.

Amino acids	Sequence	Receptor preference^a^
1–5	f-MGIIA	Formyl peptide receptor (FPR) 1
1–10	f-MGIIAGIIKF	FPR1
1–12	f-MGIIAGIIKFIK	FPR1/FPR2
1–16	f-MGIIAGIIKFIKGLIE	FPR2
**1–21 (PSMα2)**	f-MGIIAGIIKFIKGLIEKFTGK	FPR2
17–21	f-KFTGK	–
12–21	f-KGLIEKFTGK	–

*^a^Receptor preferences as described in Ref. (24)*.

*Bold font indicates the original full length peptide sequence*.

### Cytotoxicity/DNA Release Measurements with Sytox Green DNA Stain

Cells (5 × 10^4^ cells/well) in RPMI (without phenol red) and the Sytox Green DNA stain (1.25 μM; Molecular Probes) were added to black 96-well plates and incubated at 37°C and 5% CO_2_ (5). Inhibitors, PBP10 (CASLO Laboratory) or diphenyleneiodonium chloride (DPI; Sigma-Aldrich), were preincubated with cells for 10 min at 37°C and 5% CO_2_ prior to stimulation. Sytox Green fluorescence was measured at indicated time points at 485/535 nm in a Mithras LB940 (Berthold Technologies) or in a CLARIOstar plate reader (BMG Labtech).

### Measurements of Plasma Membrane Disruption by Lactate Dehydrogenase (LDH) Release

Neutrophils (2 × 10^6^ cells/ml) were incubated with PSMα2 or hexadecyltrimethylammonium bromide (CTAB, Sigma) for 10 min at 37°C. LDH was measured in the supernatants with cytotoxicity detection kit (LDH, Roche) as described in Ref. (25). Absorbance at 490 nm was measured, and the results show the percentage of maximal LDH release [Triton X-100 (TX-100)-treated samples].

### Measurements of NE Activity in Neutrophil Lysates

Isolated neutrophils from a Papillon–Lefèvre syndrome (PLS) patient and an unrelated healthy control donor were used to prepare cell lysates (5 × 10^7^ cells/ml in KRG with 0.1% TX-100) by vortexing and pelleting cellular debris by centrifugation. Supernatants were diluted 1:20, and NE activity was assessed by the fluorogenic elastase substrate MeOSuc-AAPV-AMC (Bachem, Weil am Rhein, Germany) at a concentration of 0.1 mM. A solution of only elastase substrate and KRG was used as a buffer control. Fluorescence was monitored in a CLARIOstar plate reader using an excitation wavelength of 355 ± 15 nm and emission wavelength at 405 ± 20 nm. The fluorescence intensity was measured at 5-min intervals over the course of 30 min.

### Microscopic Visualization of Cells

Neutrophils or human A375 melanoma cells (2.5 × 10^5^ cells/well) were suspended in RPMI and added to poly-lysine-coated coverslips. After stimulation with PMA (50 nM; Sigma-Aldrich) or PSMα (indicated concentrations), the cells were incubated at 37°C in the presence of 5% CO_2_ or at 4°C for indicated time. To visualize NETs, samples were fixed in 4% paraformaldehyde for 30 min at room temperature and stained with antibodies against MPO (DAKO), NE (Calbiochem), or Histone-1 (Acris) followed by secondary antibody staining. Finally, the coverslips were incubated with DAPI (1 μg/ml) for 5 min and then mounted in ProLong Gold antifade (Molecular Probes). For nuclear morphology experiments, neutrophils were incubated with PSMα2 for 10 min or with PMA for 30 min or 2 h, the cells were fixed on ice with 4% paraformaldehyde, permeabilized with cold acetone and methanol (1:1) for 5 min, and stained with SUN2 antibody (Abcam) followed by secondary antibody staining. To visualize microbes in the NETs, we used green fluorescent protein (GFP)-expressing *Candida albicans*, strain CAI4 containing a *C. albicans*-specific pENO1-GFP-CyC1t plasmid (26), or *S. aureus*, strain RN4220 containing the pCN-GFP plasmid that was a kind gift from Dr. Maria Lerm (Linköping University, Sweden). GFP-expressing *S. aureus* or *C. albicans* (10^6^ microbes/well) were added after NET formation and left for 15 min at RT or 1 h at 37°C before being processed as above. The cells were imaged either using a confocal microscope (Zeiss LSM700) with the ZEN software (Zeiss) or using an Olympus BX41 epifluorescent microscope with the cellSens software. Postprocessing was done either in the ZEN software or in the open source ImageJ image processing software and involved adjustment of brightness that was done identically in all samples within the same experiment.

### Statistical Analysis

Statistical analysis was performed on raw data using one-way analysis of variance followed by Dunnett’s multiple comparison *post hoc* test. The analysis was performed in the GraphPad Prism software (version 6.0). Statistical significant differences are expressed in the figures by *p ≤ 0.05, **p ≤ 0.01, and ***p ≤ 0.001.

## Results

### PSMα Peptides Induce Rapid NET Formation

**Figure 1:**
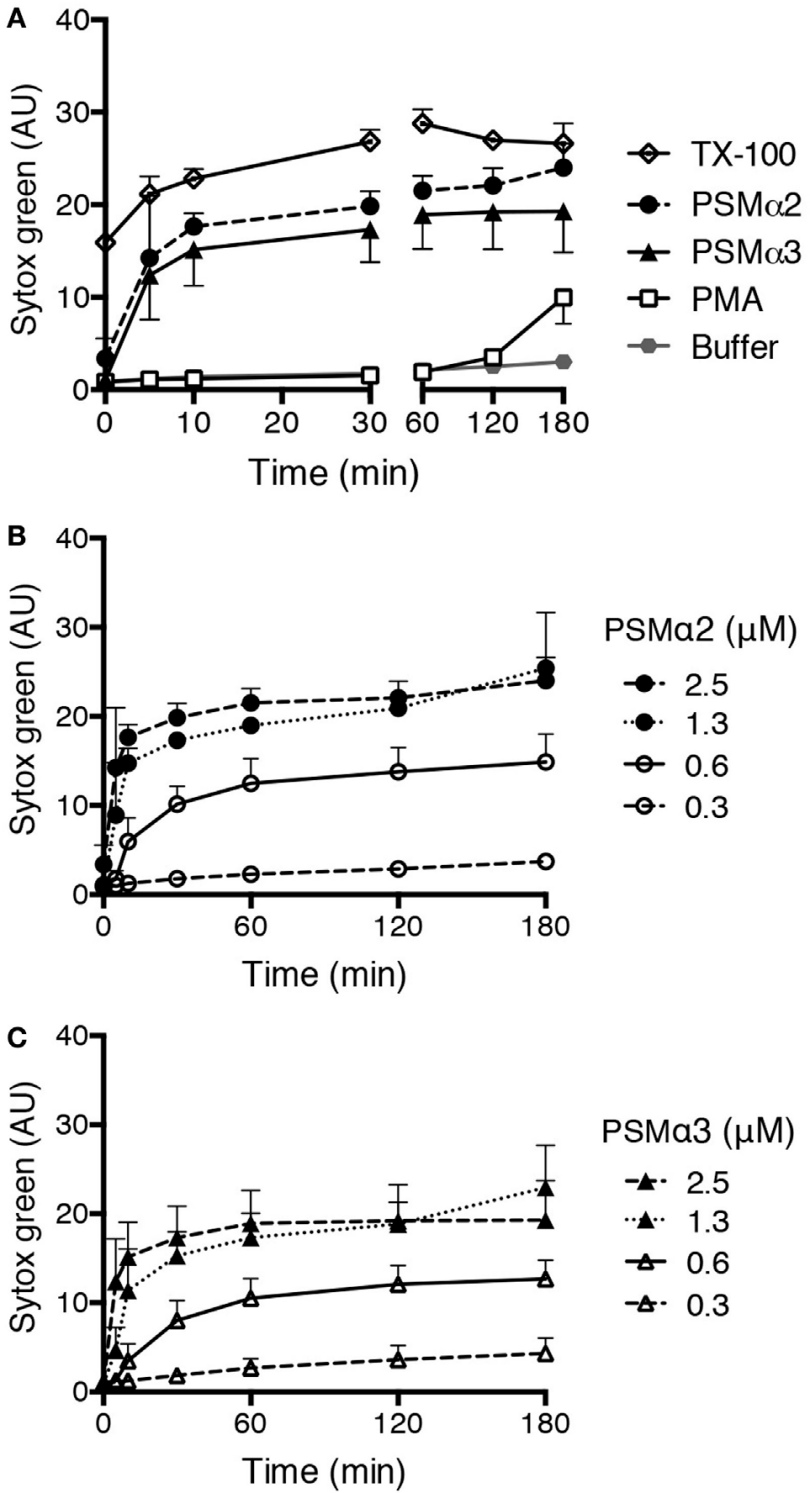
**PSMα peptides induce rapid Sytox green fluorescence in neutrophils**. **(A)** Sytox Green fluorescence measured from neutrophils incubated without or with phorbol myristate acetate (PMA) (50 nM), PSMα2 (2.5 μM), PSMα3 (2.5 μM), or Triton X-100 (TX-100; 1%). **(B)** Measurement of Sytox green fluorescence in neutrophils treated with different concentrations of PSMα2 or **(C)** PSMα3. Results are from three independent experiments shown as mean ± SD.

Formation of NETs can be measured by a Sytox green assay that evaluates the amounts of extracellular DNA. When neutrophils are treated with the common NET-inducer PMA, an increase in Sytox green fluorescence can be observed after 3 h (Figure 1A), which is in accordance with the time neutrophils takes to form NETs after PMA stimulation (7, 12). We tested PSMα2 and PSMα3 in the same system and found that they rapidly induced Sytox green fluorescence with a significant increase already after 5 min (*p* < 0.05; Figure 1A). The response occurred in a dose-dependent manner, with approximately 1 μM peptide required for full response (Figures 1B,C). Compared to PMA, the PSM peptides gave a more rapid response and the response was of higher magnitude (Figure 1A) with signals close to the 100% lysis control (neutrophils treated with the detergent TX-100).

### The PSMα-Triggered NETs Are Morphologically Indistinguishable from PMA-Induced NETs

**Figure 2:**
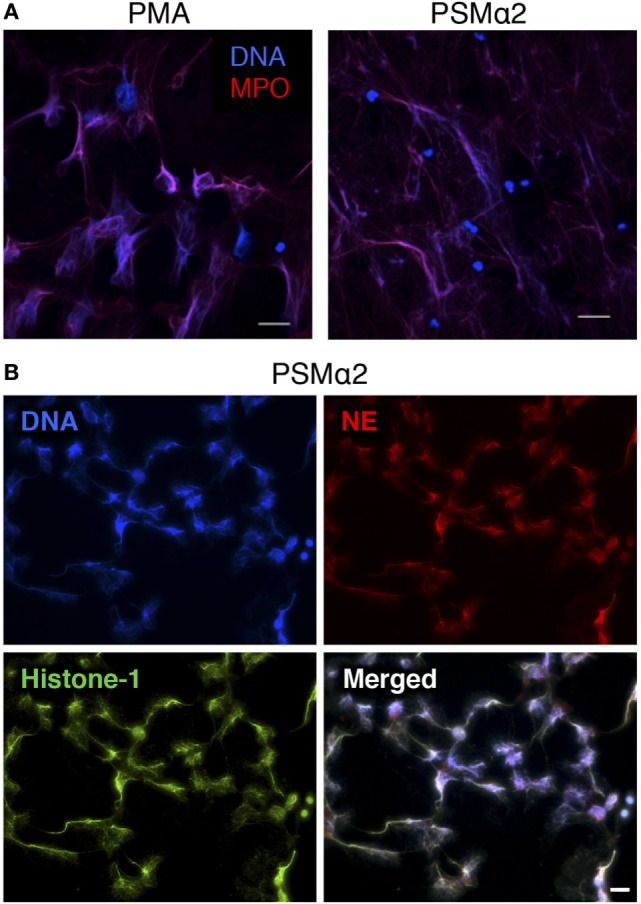
**Neutrophils treated with PSMα2 form extracellular traps**. **(A)** Neutrophils stimulated with phorbol myristate acetate (PMA) (50 nM) for 3 h or PSMα2 (2.5 μM) for 30 min were fixed and stained for DNA (blue) and MPO (red). **(B)** Neutrophils visualized after 4 h stimulation with PSMα2 (2.5 μM) after staining for DNA (blue), neutrophil elastase (NE) (red), and histone-1 (green). The cells were visualized using an epifluorescence microscope, and the scale bars represent 20 μm.

We next visualized the PSMα-treated neutrophils by immunofluorescence microscopy and found that the neutrophils had cast out DNA fibers that highly resembled the NETs induced by PMA stimulation (Figure 2A). Typical NETs are extracellular structures that consist of a chromatin backbone covered in proteins that mainly originate from intracellular granules, including MPO and NE (5). Stimulation of neutrophils with PSMα resulted in extracellular structures that contained not only DNA but also common protein markers of NETs, namely MPO (Figure 2A), NE, and Histone-1 (Figure 2B). Thus, neutrophils treated with PSMα peptides formed NETs, which were morphologically similar to classic PMA-induced NETs. However, NET release was much more rapid in response to PSMα than to PMA (Figures 1A and 2A).

**Figure 3:**
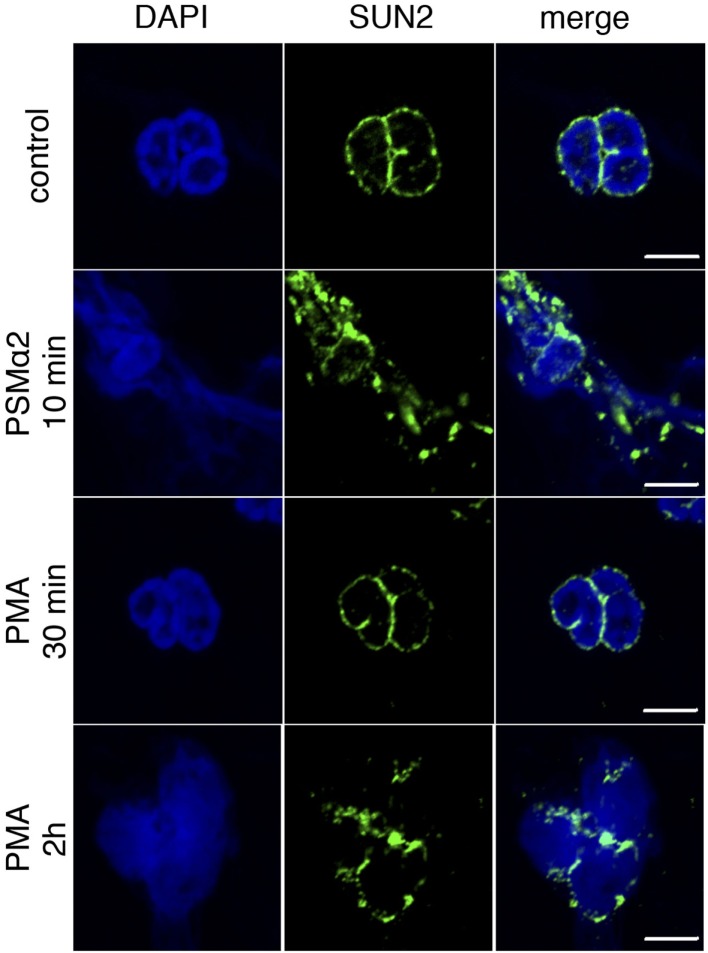
**Loss of nuclear membrane integrity after stimulation**. Neutrophils were treated with PSMα2 for 10 min or phorbol myristate acetate (PMA) for 30 min or 2 h after which the cells were fixed and stained for the nuclear envelope protein SUN2 (green) and DNA (blue). The cells were visualized in a confocal microscope. The images are representative of three independent experiments. The scale bars represent 5 μm.

The rapidness of NET formation after PSMα stimulation was also evident when monitoring nuclear morphology. In naive cells, the nuclear envelope marker SUN2 showed a distinct perinuclear staining pattern, but disrupted nuclear membranes were observed after short incubation (5–10 min) with PSMα2. With PMA, disruption of nuclear membranes was a much slower process and observed first after 2-h stimulation (Figure 3).

### PSMα-Induced NETs Capture *S. aureus* and *C. albicans*

**Figure 4:**
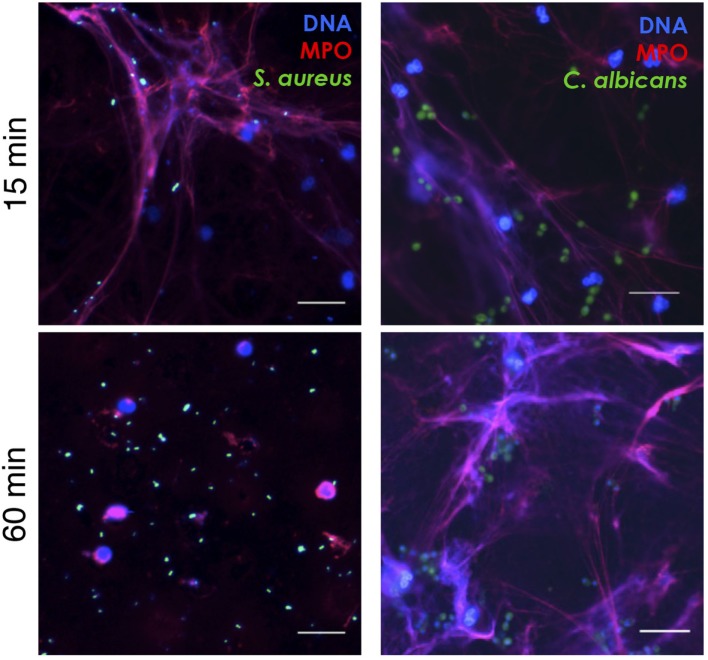
**PSMα2-induced NETs capture *Staphylococcus aureus* and *Candida albicans***. NET formation was induced by PSMα2 for 3 h after which green fluorescent protein-expressing *S. aureus* or *C. albicans* were added to the NETs at a multiplicity of infection (microbes:neutrophils) 4:1 for either 15 min RT or 1 h 37°C. The samples were fixed and stained for DNA (blue) and MPO (red). Shown is one representative experiment from at least three independent experiments performed. The cells were visualized using an epifluorescence microscope, and the scale bars represent 20 μm.

Neutrophil extracellular traps are believed to function as a defense system that can capture and kill microbes extracellularly, and microbial expression of DNase has been shown to confer relative resistance toward NET-mediated capture by degradation of the DNA fibers (27, 28). We added GFP-labeled *S. aureus* (expressing DNase; Figure S1 in Supplementary Material) and *C. albicans* (lacking DNase; Figure S1 in Supplementary Material) to analyze the binding of microbes to PSMα2-induced NETs. Shortly after addition of the labeled microbes to NETs, both *S. aureus* and *C. albicans* were frequently found attached to NET structures (Figure 4, upper). However, when the microbes were left on the NET structures for 1 h, *S. aureus* caused disintegration of the NETs, and most bacteria were found in free forms, not bound to DNA (Figure 4, lower). In contrast, prolonged incubation with *C. albicans* did not result in disintegration of the NETs (Figure 4, lower). The microbes bound similarly to PMA-induced NET structures, and the NETs were also degraded with time by *S. aureus* but not *C. albicans* (data not shown). This demonstrates that PSMα-induced NETs contain the same components as PMA-induced NETs (Figure 2) and more importantly that they are able to catch (at least DNase negative) microbes.

### PSMα-Induced NET Formation Is Not Dependent on FPR2

**Figure 5:**
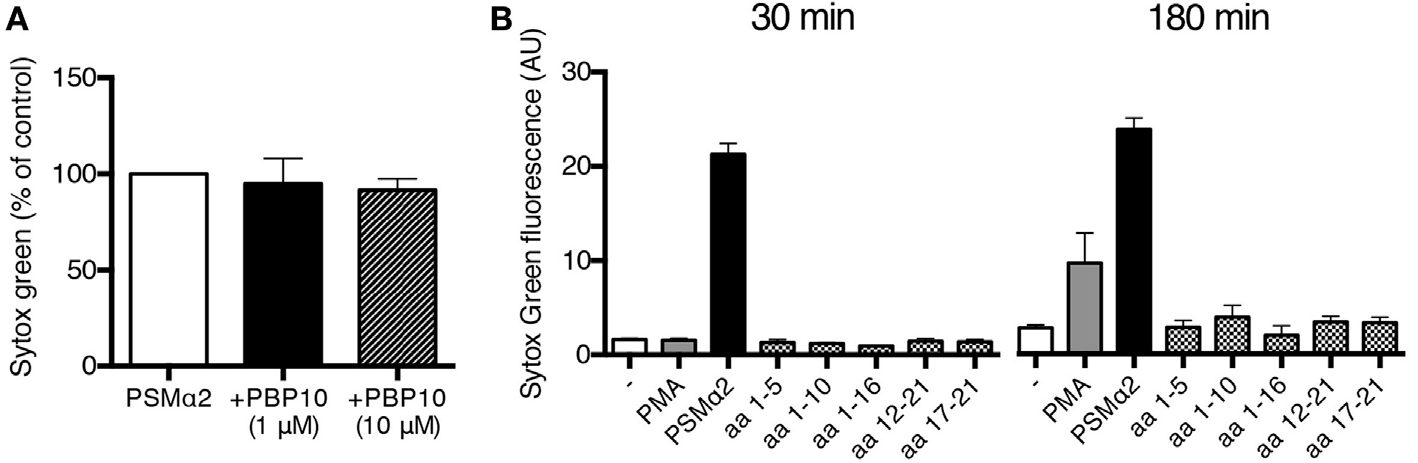
**PSMα2-induced NET formation is not dependent on FPR2**. **(A)** NET formation measured by Sytox Green fluorescence from neutrophils treated with PSMα2 (2.5 μM) for 30 min in the absence or presence of indicated concentrations of the FPR2 inhibitor PBP10. Results from three independent experiments are presented as percent of PSMα2-treated cells ±SD. **(B)** Neutrophils incubated for 30 or 180 min in the absence or presence of phorbol myristate acetate (PMA) (50 nM), PSMα2 (5 μM), or shorter variants of the PSMα2 peptide (5 μM). The amino acids (aa) of the incomplete PSMα2 peptides are shown, see also Table 1. Results show the mean ± SD from three independent experiments.

PSMα2 has been shown to have various activating effects on neutrophils through binding to the chemoattractant receptor FPR2 (21, 22). To further characterize the NET formation induced by PSMα peptides, we investigated the possible involvement of FPR2. The specific FPR2 inhibitor PBP10, at doses that completely abrogated FPR2-mediated activation of neutrophils [(29); and not shown], had no effect on PSMα2-induced NET formation (Figure 5A) indicating that FPR2 signaling is not necessary for PSMα2-triggered NET formation. We also made use of a collection of shorter PSMα2 peptide variants (Table 1) differing in FPR2 specificity; some of the shorter variants activate neutrophils through FPR2, some shift receptor preference to another receptor of the formyl peptide receptor family, FPR1, and others are inactive [Table 1; (24)]. None of the shorter PSMα2 variants induced NET formation, neither after 30 nor 180 min incubation (Figure 5B). These results indicate that FPR2 is neither necessary nor sufficient for PSMα2-induced NET formation and that only the full-length peptide is capable of triggering NET formation.

### PSMα-Induced NET Formation Is Independent of MPO, ROS, and NE

**Figure 6:**
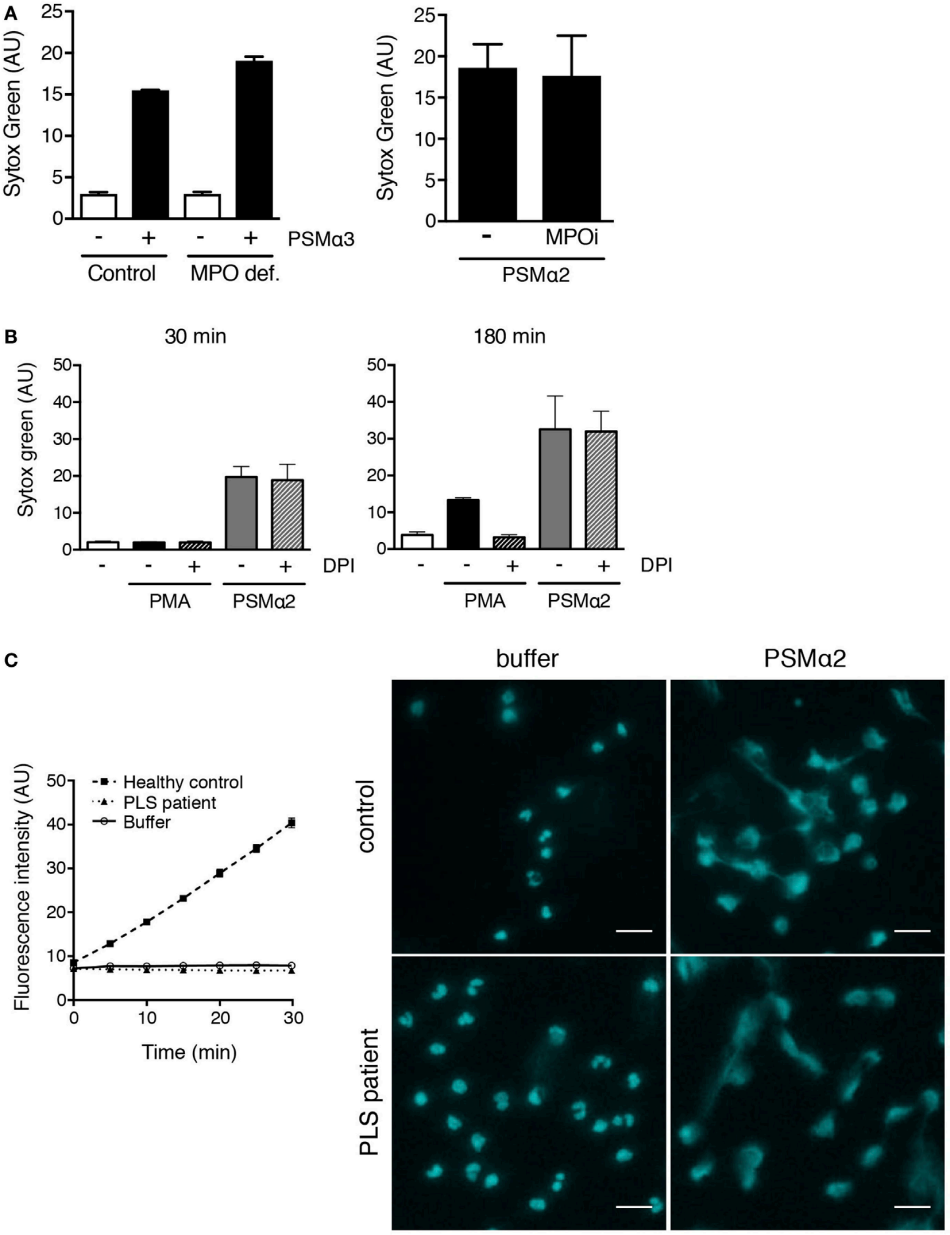
**PSMα2-induced NET formation is independent of MPO, ROS, and NE**. **(A)** Neutrophils from an individual with complete MPO deficiency (10) and a healthy control donor stimulated with PSMα3 (2.5 μM) for 30 min. Results from one experiment are shown as mean ± SD of triplicate samples (left figure). Neutrophils from healthy control donors stimulated with PSMα2 (2.5 μM) in the absence or presence of MPO inhibitor AZM198 (MPOi; 10 μM). Results are shown as mean ± SD from two independent experiments after 30-min stimulation (right figure). **(B)** Neutrophils stimulated with phorbol myristate acetate (PMA) (50 nM) or PSMα2 (2.5 μM) in the absence or presence of the NADPH oxidase inhibitor DPI (10 μM). Results are shown as mean ± SD of three independent experiments. **(C)** Neutrophil lysates from one PLS patient (dotted line) and one unrelated healthy control (dashed line) were tested for NE activity using a fluorogenic substrate (left). A buffer control (solid line) without any cell lysate is shown for comparison. Neutrophils from the PLS patient and the control were either incubated with buffer or PSMα2 (2.5 μM) for 1 h. The cells were stained for DNA (pseudocolored cyan) and visualized using an epifluorescence microscope (right); scale bars represent 20 μm.

The formation of NETs in response to PMA and microbes (8) has been shown to be dependent on the azurophil granule enzyme MPO by that neutrophils from individuals deficient in protein do not form NETs (8). Further, we have shown that enzymatic conversion of ROS by MPO inside granules is a prerequisite for PMA-induced NET formation (10). We thus investigated whether MPO was required for PSMα-induced NET formation by using neutrophils from a completely MPO-deficient donor [previously described in Ref. (10)] that was sampled at two occasions (with nearly identical results). Neutrophils from this donor were unable to form NETs in response to PMA (10), but readily formed NETs in response to PSMα (Figure 6A). Furthermore, PSMα2-induced NET formation was not affected by the specific MPO inhibitor AZM198 (Figure 6A) at doses that completely block PMA-induced NETosis (10).

The role of MPO is to catalyze reactions involving ROS generated by the NADPH oxidase, and this electron transfer system also needs to be operational in order for neutrophils to form NETs in response to PMA (7, 10). In line with the finding that MPO was not needed for PSMα-induced NET formation (Figure 6A), we also found that the NADPH oxidase inhibitor DPI was without effect on PSMα-induced NETs, while it potently inhibited PMA-induced NET formation (Figure 6B). Thus, PSMα-induced NET formation is independent of NADPH oxidase-derived ROS and MPO activity.

The serine protease NE has been shown to be of involved in the process leading to NETosis (11, 30). We, therefore, tested whether NE was needed for PSMα-induced NET formation by using neutrophils from a patient with Papillon-Lefèvre syndrome (PLS), a condition where neutrophil serine protease activity is missing, including NE activity (30) (Figure 6C). PLS neutrophils formed NETs in response to PSMα (Figure 6C). Thus, in contrast to PMA- and fungi-induced NET formation (11, 30), NE activity seems to be dispensable for PSMα-induced NET formation.

### PSMα-Induces NET Formation at 4°C

**Figure 7:**
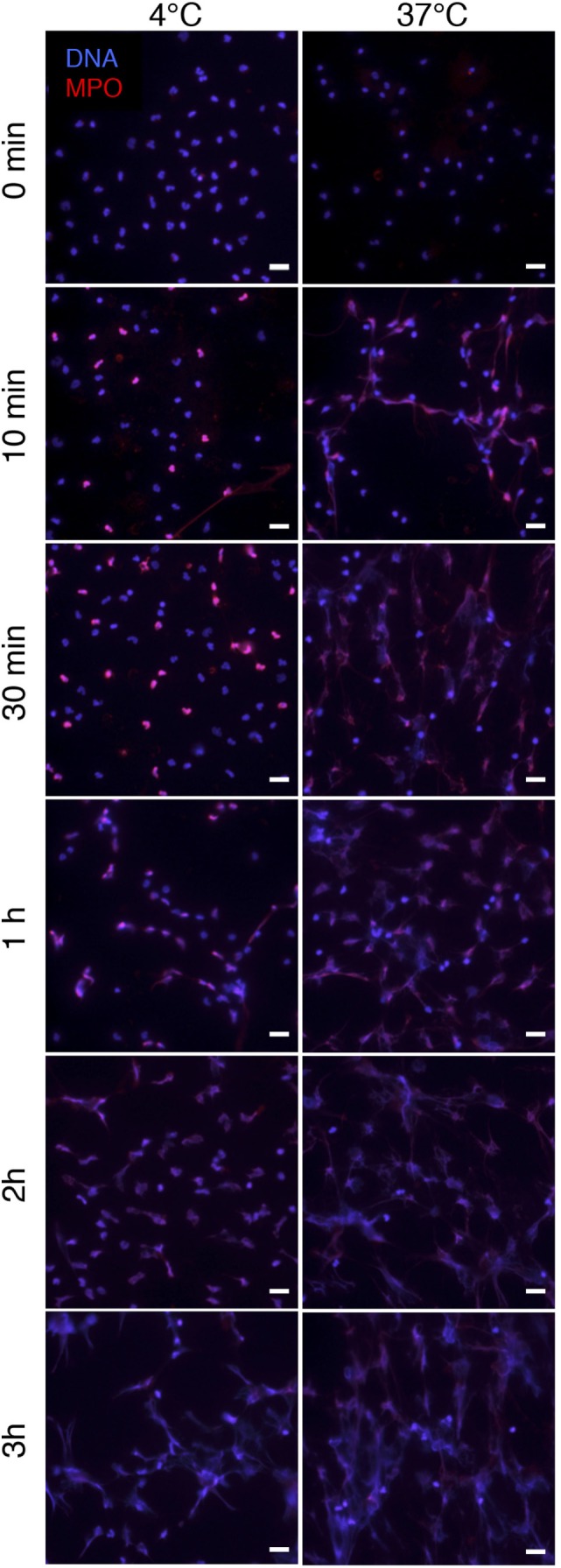
**PSMα2-induced NET formation occurs at 4°C**. Neutrophils were treated with PSMα2 (2.5 μM) and incubated at 4°C or 37°C. At the indicated time points after stimulation, neutrophils were fixed and stained for DNA (blue) and MPO (red). The images are from one representative experiment of three independent experiments performed. The cells were visualized using an epifluorescence microscope, and the scale bar represents 20 μm.

Since PSMα-triggered NET formation did not involve FPR2, NADPH oxidase, or MPO, we next assayed NET formation at cold temperatures to test whether active cell signaling was at all required. At 4°C, PSMα still triggered NET formation, although the process was slower than at 37°C (Figure 7).

### Disruption of the Plasma Membrane Is Not Sufficient to Trigger NET Formation

**Figure 8:**
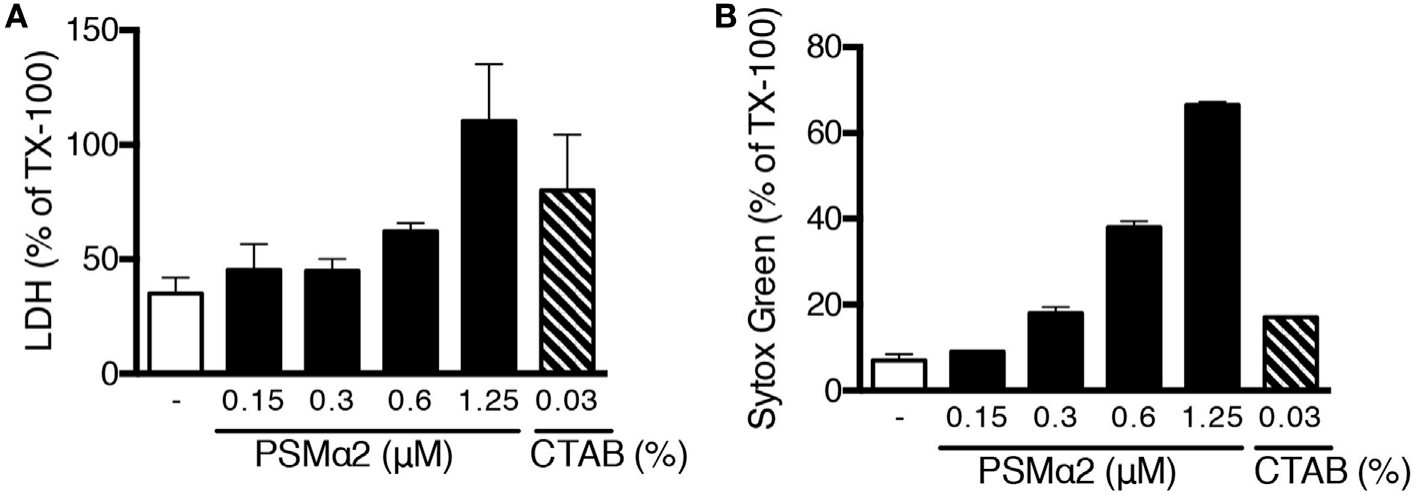
**Plasma membrane disruption is not sufficient to induce NET formation**. **(A)** Neutrophils were stimulated for 10 min with PSMα2 or the detergent CTAB after which lactate dehydrogenase (LDH) was measured in the supernatants. **(B)** Sytox Green fluorescence in neutrophils treated with PSMα2 or CTAB for 10 min. Results are from two independent experiments, performed in triplicates, and shown as mean ± SD in % or max (=Triton X-treated samples).

To gain further insights into the mechanisms whereby PSMα peptides mediate NETs release and to test whether permeabilization/disruption of neutrophil plasma membranes is sufficient to trigger NETs release, we compared the effects of PSMα2 with those of the cationic detergent CTAB. For PSMα2, Sytox green staining correlated well with the release of cytosolic LDH (Figure 8A), the latter being a clear sign of plasma membrane disruption. In contrast, CTAB treatment resulted in robust disruption of plasma membranes with close to maximal LDH release, but did not cause NET formation (Figure 8B). These data demonstrate that disruption of the plasma membrane is not sufficient to cause NET formation.

### PSMα Peptides Permeabilize Non-Myeloid Cells without Extracellular DNA Release

**Figure 9:**
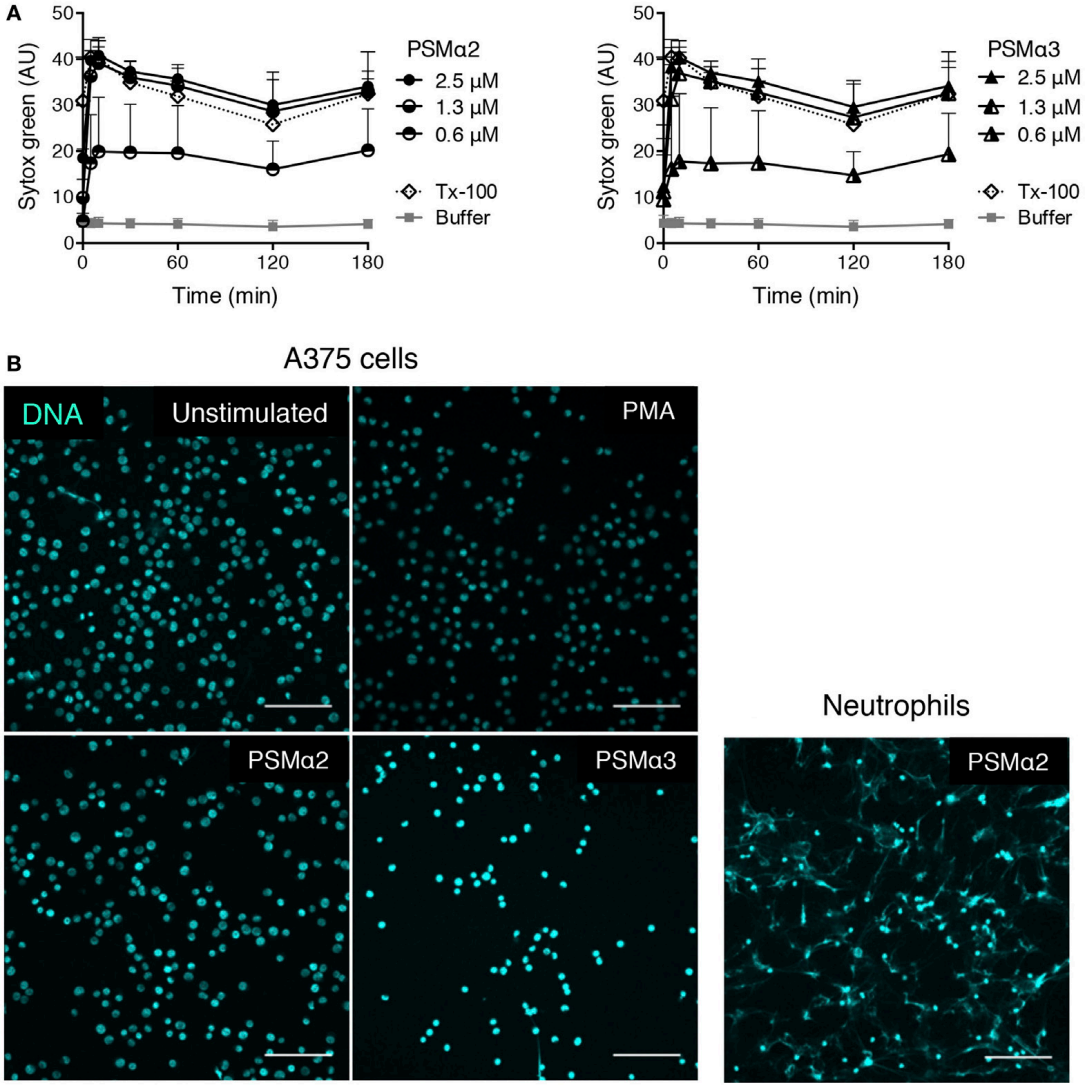
**PSMα2 and PSMα3 on A375 melanoma cells**. **(A)** Measurement of Sytox Green in A375 melanoma cells stimulated with different concentrations of PSMα2 or PSMα3. Results from three independent experiments are shown as mean ± SD. **(B)** Micrographs of A375 melanoma cells, untreated, stimulated with PSMα2 or PSMα3 (2.5 μM) for 1 h, or PMA (50 nM) for 3 h. For comparison, neutrophils stimulated with PSMα2 (2.5 μM) for 1 h are shown to the right. All samples were fixed and stained for DNA (DAPI, pseudocolored for clarity). The cells were visualized using a confocal microscope, and the scale bars represent 100 μm. Images are representative of three independent experiments performed.

The PSMα peptides are alpha-helical in structure and are amphipathic, features that are common for pore-forming peptides (20, 31). Theoretically, such peptides have the possibility to permeabilize membranes of all cell types. To establish whether PSMα-induced formation of extracellular traps is something unique to neutrophils, we tested the effect of PSMα peptides on unrelated non-myeloid cells, using a melanoma cell line. At similar concentrations as those needed induce extracellular trap formation in neutrophils (Figure 1), the PSMα peptides caused a rapid increase in DNA staining of the melanoma cells when analyzed in the Sytox green assay (Figure 9A). However, when the PSMα-treated melanoma cells were visualized, it was clear that the DNA staining originated from intracellular nuclei and that extracellular DNA fibers were not present (Figure 9B). As expected, PMA stimulation of melanoma cells also did not induce any DNA expulsion. This indicates that the PSMα peptides are cytotoxic to cells other than neutrophils, but that the cytotoxicity is not associated with the formation of extracellular DNA protrusions in all cell types.

## Discussion

In this study, we show that PSMα peptides secreted from CA-MRSA, aggressive *S. aureus* strains, induce NET formation. The NETs triggered by PSMα peptides were morphologically comparable with NETs induced by PMA and contained typical NETs markers such as DNA, histone, MPO, and NE. Furthermore, we found that PSMα-induced NETs were functional and capable of capturing microbes in a manner similar to PMA-induced NETs. Interestingly, although we found *S. aureus* to be initially trapped in the NETs, they also had the ability to break free, presumably due to secretion of DNase. The secretion of DNase is a virulence determinant of *S. aureus* that has been described to facilitate escape from NETs and to contribute to pathogenesis by permitting invasion of deeper organs (28, 32). CA-MRSA strains typically express DNase (33). The fungus *C. albicans* was also found to become trapped in the PSMα-induced NETs and could not break free, which is perfectly in line with the lack of DNase expression (Figure S1 in Supplementary Material).

Although PSMα-induced NETs were morphologically and functionally similar to the classic PMA-induced NETs, the mechanism behind their formation seems to be entirely different. Typically, NET formation is preceded by a regulated sequence of cellular events (e.g., vacuolization of the cytoplasm, rapid chromatin decondensation, and breakdown of nuclear and granular membranes) resulting in a distinct type of regulated cell death, NETosis (34). Such classic NET formation is triggered not only by the PKC activator PMA but also by various microbes (7, 12, 35), and it is dependent on ROS formation from the NADPH oxidase and on the activity of neutrophil enzymes such as MPO, NE, and PAD4 (8, 11, 36). We previously demonstrated that ROS need to be further modified by MPO at an intracellular location (10), presumably an organelle formed by heterotypic fusion of specific and azurophil granules (37), in order for PMA-triggered NET formation to occur. Despite the central position of ROS, also ROS-independent modes of NET formation have been described (12, 38), and although ROS-independent NET formation can be faster than classic ROS dependent (where extracellular DNA is typically not seen until after 2–3 h), this process also seem to require active signaling (15, 38). Further, even faster NET formation has been observed, e.g., in response to *S. aureus* (15, 16), an effect most likely mediated by secreted bacterial factors such as the pore-forming toxin Panton–Valentine leukocidin (16) and leukotoxin GH (19). These two toxins have been shown to release NETs in different manners. Panton-Valentine leukocidin was shown to induce vital NETosis by budding off nuclear-filled vesicles that burst once outside the neutrophils to form NETs (16). Leukotoxin GH was on the other hand shown to induce NET formation by its pore-forming action, and the process resulted in neutrophil cell death (19).

Toxins from other bacteria have also been found to be responsible for the formation of extracellular traps, in both monocytes/macrophages and neutrophils. Some of those processes are ROS dependent, such as those induced by leukotoxin from *Mannheimia haemolytica* (39) and pyocyanin toxin from *Pseudomonas aeruginosa* (40), whereas other NET-inducing toxins have been described but the mechanism is unknown such as for hemolysin from *Escherichia coli* (39), ArgD from *S. aureus* (41), and M1 protein from *Streptococcus pyogenes* (42).

It is clear that the formation of NETs can be the final outcome of numerous different mechanisms and that different agents trigger NET formation differently. Here, we show that PSMα induce suicidal NET formation that is distinct from the classic NET formation in that it is a very rapid process, with NETs being observed after only 5–10 min stimulation. Furthermore, this NET formation was independent of NADPH oxidase-derived ROS, MPO, and NE activity.

The PSMα peptides are part of a toxin family that is emerging as key virulence factors of highly aggressive CA-MRSA isolates (43). These alpha-helical peptide toxins are known not only to attract and activate neutrophils by ligation of the chemotactic receptor FPR2 (21, 22) but also to lyse these cells at higher (low micromolar) concentrations (20). Our data clearly demonstrate that FPR2, although capable of recognizing PSMα peptides with high affinity, was not involved in the NETs induction: inhibition of FPR2 had no effect on PSMα-induced NET formation and shorter PSMα variants did not trigger NET release even though some of these variants activate FPR2 (24). Importantly, none of the shorter variants possess the ability to permeabilize apoptotic cell membranes (24), suggesting that NET formation is a direct consequence of the membrane-disturbing effects of the peptides. It is possible that the shorter peptides do not form the alpha-helical secondary structure that is important for the membrane insertion and disturbance (44). As far as we are aware, *in vivo* data on concentrations of PSMα2 during CA-MRSA infections are lacking. However, it is clear that these peptide toxins represent major secreted products by the bacteria and that the level of PSM release corresponds to the virulence of a particular strain (20, 45). The concentrations needed to induce NET formation *in vitro* are in the high nanomolar to low micromolar range, i.e., higher than the doses needed to, e.g., activate chemotactic migration *via* FPR2 (21). This could be an example of the intricate interplay between bacteria and host immunity in that lower levels of PSM peptides recruit neutrophils to the site of infection *via* FPR2 and that higher levels (e.g., close to the site of infection and/or high numbers of bacteria) instead cause NET formation.

At this moment, we can only speculate whether the NET formation we describe here is an active response launched by neutrophils or merely a passive outcome of membrane disturbances. However, several results support the latter: NET formation in response to PSMα peptides was remarkably fast and did not require NADPH oxidase-derived ROS, MPO activity, NE activity, or FPR2 signaling. Also, PSMα-induced NET formation proceeded at 4°C, even though the process was not as rapid as at 37°C. Cell membranes are more rigid at colder temperatures, and the delay in NET formation would be compatible with the idea that membrane disturbances evoked by the PSMα peptides initiate the NETs expulsion. A non-programmed NET formation is in line with what was shown for leukotoxin GH, as mentioned above, and the authors also established that NETs can in fact be released as a consequence of non-specific membrane damage such as that evoked by electropermeabilization (19). A recent study demonstrated that small non-polar nanoparticles damage plasma membranes as well as lysosomal membranes of neutrophils, which result in NET formation (46). In line with this, we found that detergent-induced disruption of plasma membranes was not sufficient to trigger NET formation, indicating that damage of other cellular membranes, e.g., nuclear and/or granule/lysosome membranes, may be required for NETs release. Since granule constituents (e.g., MPO and NE) are found evenly dispersed on the DNA fibers (Figure 2B), this indicates that at least azurophil granule membranes are disrupted during PSMα-induced NET formation. Although we cannot draw any conclusions as to the causality (if any) between lysosomal rupture and NETs release, preliminary data indicate that LAMP-2 staining was disturbed after PSMα treatment, implying that LAMP-2-expressing lysosomes are also damaged by the peptides.

The PSMα peptides were capable of permeabilizing also membranes of non-myeloid cells, but here cell death occurred without protrusion of extracellular DNA fibers. This shows that the discharge of DNA structures as a result of membrane disruption does not occur for all cell types, and neutrophils clearly possesses some unique characteristic that enables DNA expulsion. Neutrophils have a very distinct nuclear morphology; it is segmented into three to four lobes and has been shown to be very malleable, a feature thought to promote fast and easy transmigration over the vessel walls (47). It is interesting to speculate that this or some other unique feature of the peculiar neutrophil nucleus (48) allows for DNA to be discharged after disintegration of the plasma and/or nuclear membranes.

In conclusion, we show that cytotoxic and membrane-disturbing PSMα peptides secreted from CA-MRSA induce rapid NET formation that is distinct from the traditional NETosis program induced by PMA or whole microbes. Although NETs are considered to be a host defense mechanism, the ability of NETs to entangle microbes depends on intact chromatin structure and is lost if subjected to DNase digestion. Thus, DNase-expressing CA-MRSA could use PSMα peptides to induce rapid NET formation as a means to kill the phagocytes without risk of being permanently immobilized in the NETs.

## Ethics Statement

This study was carried out in accordance with the recommendations of the Regional Ethical Review Board in Gothenburg, Sweden, with written informed consent from all subjects. All subjects gave written informed consent in accordance with the Declaration of Helsinki. The protocol was approved by the Regional Ethical Review Board in Gothenburg, Sweden.

## Author Contributions

Design of the work: HB, AW, KC, CU, HF, CD, AK, and JB. Performance of experiments: HB, AR, FK, JE, AW, and MS. Analysis and interpretation of data: HB, AR, FK, JE, AW, MS, and JB. Writing of manuscript: HB and JB.

## Conflict of Interest Statement

The authors declare that the research was conducted in the absence of any commercial or financial relationships that could be construed as a potential conflict of interest.
