# Supplementary figures and images for: Phenol-Soluble Modulin α Peptide Toxins from Aggressive Staphylococcus aureus Induce Rapid Formation of Neutrophil Extracellular Traps through a Reactive Oxygen Species-Independent Pathway
Source: Front Immunol. 2017 Mar 9;8:257. doi: 10.3389/fimmu.2017.00257 (PMC5343011; doi:10.3389/fimmu.2017.00257)

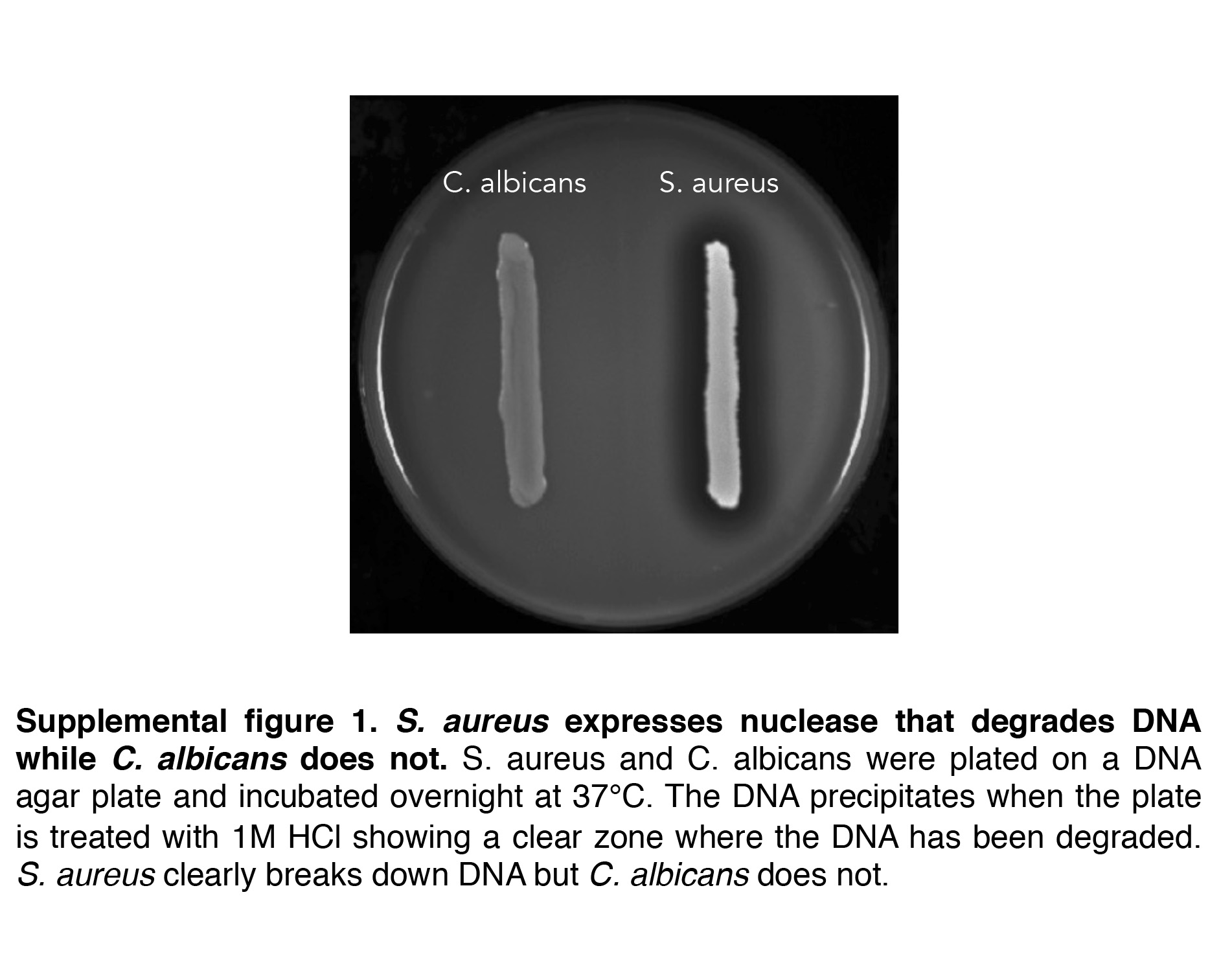

Supplement: Supplementary file 1 [file Image_1.JPEG]
